# Insect Innate Immunity Database (IIID): An Annotation Tool for Identifying Immune Genes in Insect Genomes

**DOI:** 10.1371/journal.pone.0045125

**Published:** 2012-09-12

**Authors:** Robert M. Brucker, Lisa J. Funkhouser, Shefali Setia, Rini Pauly, Seth R. Bordenstein

**Affiliations:** Department of Biological Sciences, Vanderbilt University, Nashville, Tennessee, United States of America; University of Cyprus, Cyprus

## Abstract

The innate immune system is an ancient component of host defense. Since innate immunity pathways are well conserved throughout many eukaryotes, immune genes in model animals can be used to putatively identify homologous genes in newly sequenced genomes of non-model organisms. With the initiation of the “i5k” project, which aims to sequence 5,000 insect genomes by 2016, many novel insect genomes will soon become publicly available, yet few annotation resources are currently available for insects. Thus, we developed an online tool called the Insect Innate Immunity Database (IIID) to provide an open access resource for insect immunity and comparative biology research (http://www.vanderbilt.edu/IIID). The database provides users with simple exploratory tools to search the immune repertoires of five insect models (including *Nasonia*), spanning three orders, for specific immunity genes or genes within a particular immunity pathway. As a proof of principle, we used an initial database with only four insect models to annotate potential immune genes in the parasitoid wasp genus *Nasonia*. Results specify 306 putative immune genes in the genomes of *N. vitripennis* and its two sister species *N. giraulti* and *N. longicornis*. Of these genes, 146 were not found in previous annotations of *Nasonia* immunity genes. Combining these newly identified immune genes with those in previous annotations, *Nasonia* possess 489 putative immunity genes, the largest immune repertoire found in insects to date. While these computational predictions need to be complemented with functional studies, the IIID database can help initiate and augment annotations of the immune system in the plethora of insect genomes that will soon become available.

## Introduction

The innate immune system evolved early in the evolution of multicellular life, while the adaptive immune system evolved in the ancestor of the vertebrate lineage [1]. Thus, in insects and other invertebrates, the innate immune system not only combats foreign invaders, but it is also employed in wound healing, stress responses, and the management of microbial symbiont populations [2]. The versatility of the insect innate immune response is in part championed by the ability of insects to colonize diverse ecological niches across the planet while defending against pathogens that inhabit those niches [3]. Indeed, immunity genes in general evolve at a faster rate than the genome as a whole [4], which is in part explained by the persistent selective pressures posed by a flux of new pathogens.

With the advent and growth of next-generation sequencing technology, rapid genome sequencing of non-model organisms is now feasible. The “i5k” initiative, launched in 2011, aims to sequence 5,000 insect genomes by 2016 [5], generating vast amounts of data for comparative studies among insects. Annotation of immunity genes in these novel insect genomes will not only provide valuable insight into the diverse mechanisms insects employ for defense, but may also contribute to the development of new insecticides for the control of agricultural pests. To facilitate the annotation of immunity genes in insects, including our own model system of *Nasonia* parasitoid wasps, we have generated an open-access database called the Insect Innate Immunity Database (IIID, http://www.vanderbilt.edu/IIID) to serve as a starting point for researchers interested in using comparative biology to identify potential immune genes in insects. The database contains the immune repertoires of five insect models (including *Nasonia*) that span several orders, and each gene is categorized based on the pathway it participates in and the role it plays in that pathway. The intuitive web interface allows researchers to search for specific immunity genes by name, retrieve all immunity genes in the database for a particular species, pathway or class, and find putative homologs for a gene of interest using an internal BLAST tool.

The jewel wasp *Nasonia* is a genus of haplodiploid, parasitoid wasps composed of four closely related species (Order: Hymenoptera): *N. vitripennis, N. giraulti, N. longicornis*, and *N. oneida*. *Nasonia* is a model system to study the genetics of interspecific differences including host-microbe interactions [6]–[8], development [9]–[11], and behavior [12]–[15]. Recently, the genomes of the first three species mentioned above were sequenced [16]. An initial characterization of immune genes in *N. vitripennis* was conducted as part of the *Nasonia* genome project [16] using two sets of Hidden Markov Models (HMMs). The first set of HMMs was generated based on alignments of select immune-related protein families from *Aedes aegypti, Anopheles gambiae* and *Drosophila melanogaster* [17], and the second set was compiled using *A. aegypti* immune genes as seeds to find orthologous genes from five vertebrate and five insect species [16]. Scanning the *N. vitripennis* gene set with these HMMs produced a total of 270 putative immunity genes (http://cegg.unige.ch/nasonia_genome). This number is likely an underestimate given that not all immune genes from the three Dipteran species above were used to generate the first set of HMMs. The second set of HMMs expanded the number of species incorporated in the models but only for those immune genes present in *A. aegypti*. Furthermore, only the *N. vitripennis* genome was examined; no study has attempted to identify immune genes in the sequenced sister species, *N. giraulti* and *N. longicornis*. Using the genes within the IIID to perform homology searches against the *Nasonia* genomes, we independently describe 306 putative immune genes in each of the *Nasonia* species, of which 146 genes were not found in previous annotations of *N. vitripennis* [16].

## Methods

### Initial Construction of the IIID

To facilitate the annotation of innate immunity genes in insects, we initially created an Insect Immunity Database (IIID) composed of the published immune repertoires of four insect models spanning several different orders: *Drosophila melanogaster,* Diptera [18], [19], *Anopheles gambiae*, Diptera [16], [20], *Apis mellifera*, Hymenoptera [17], [21], and *Acrythosiphon pisum*, Hemiptera [22]. Our criteria for inclusion were that the species have a complete, publicly-available genome sequence, that the innate immune genes have been previously identified in computational or molecular studies, and that each species has an extensive review of its global immune pathways available as a resource. Sequence information was obtained through NCBI for the 105 immunity genes described for *Acrythosiphon pisum* [22], 317 genes for *Anopheles gambiae* [20], [23], 379 genes for *Drosophila melanogaster* [18], [19], and 174 genes for *Apis mellifera* [17], [21]. In total, 975 genes were included in the dataset used to analyze the *Nasonia* genomes. Each gene was categorized into its primary, secondary and tertiary pathways of putative function (i.e. Toll pathway, IMD pathway, humoral response, JAK/STAT, and cell cycle regulation) and into finite classes of function based upon its putative role in an immune response. Such classes include recognition (identifying potential pathogens and stressors), signaling (communicating between recognition and response), and response (molecules that interact with the pathogen or stressor).

### Comparative Analysis of N. Vitripennis Immunity Genes

To validate the utility of this database, we used a sequence similarity BLASTx approach to mine for putative homologs of the 975 protein sequences in the IIID within the *N. vitripennis* transcriptome (OGS v1.2). A total of 18,941 unique transcripts were obtained from NasoniaBase (http://hymenopteragenome.org/nasonia/). For the BLASTx analyses, we used the BLOSUM62 matrix with a word size of 3 and a gap cost of 11, −1. The results were filtered to only contain hits with an *E-*value <1e-10, a bit score ≥30,. A total of 1206 *N. vitripennis* transcripts were similar to entries in the IIID (Table S1). To eliminate redundancies in the dataset, a reciprocal BLASTx analysis for each of the 1206 *Nasonia* transcripts was conducted against each of the four insect immunity gene datasets. This analysis resulted in 306 unique immune gene identifiers in *Nasonia vitripennis* (Table S2).

### Analysis of N. Giraulti and N. Longicornis Immunity Genes

Since the immune genes in the sister species *N. giraulti* or *N. longicornis* had not yet been evaluated, we conducted independent BLASTn analyses of the 489 *N. vitripennis* immunity genes (IIID predictions and previously annotated immune genes) against the *N. longicornis* (NCBI assembly name Nlon_1.0) and *N. giraulti* (NCBI assembly name Ngir_1.0) scaffolds [16]. The parameters for the BLASTn search are as follows: *E-*value <1e-10, word size 11, low complexity filter, and a gap cost 5, −2. For each species, best hits for the 489 genes were manually assessed as to the *E*-value and bit score, as previously described above, and nucleotide sequences were compiled for each gene in *N. giraulti* (Table S3) and *N. longicornis* (Table S4).

## Results

The initial IIID was compiled using the immune repertoires of *D. melanogaster*, *A. gambiae*, *A. pisum*, and *A. mellifera* for a combined total of 975 genes. Using this dataset to perform homology searches against the *N. vitripennis* transcriptome, we identified 306 putative immune genes. 138 of these genes were previously reported as immune genes in the *Nasonia* genome (Nvit_1.2) paper, which identified a total of 270 putative immune genes using HMMs for protein domains common in immunity gene families [16]. We also manually searched the *N. vitripennis* official gene set (v1.2) and the *Nasonia* literature [24]–[26] for genes with annotations similar to those of conserved immunity genes in other insect species. In total, we found 66 genes from our manual search that were not reported in Werren *et al.,* [16]. Importantly, 146 of the 306 genes identified using the IIID were not previously described in any of the *Nasonia* literature. Furthermore, using the IIID, we were able to assign names to 28 genes that were not previously annotated in the *N. vitripennis* gene set (Nvit_1.2). Conversely, a total of 183 immune genes identified previously in the *Nasonia* literature are absent from the IIID analyses of the *N. vitripennis* genome (see discussion).

Combining the immune genes identified using the IIID with the additional genes described in the literature, *N. vitripennis* possesses a total of 489 putative immunity genes (Table S2). This is the largest predicted immune repertoire found in insects to date. None of the genes found in *N. vitripennis* were missing in either *N. giraulti* (Table S3) or *N. longicornis* (Table S4).

## Discussion

Using the IIID, we increased the putative *Nasonia* immune repertoire by 58% in comparison to the number of immune genes originally published in the *Nasonia* genomes [16], while only finding 46% of the immune genes originally published. The missing genes are of interest. It is important to note that the *Nasonia* immune gene set in the genome sequence [16] was identified using Hidden Markov Models (HMMs) that search for genes with protein domains common in immunity genes. One problem with this approach is that all members of a gene family with an immunity-related protein domain may not have a biological role in innate immunity if this domain can also function in other processes. Thus, using only HMMs to find immune genes will increase the likelihood of false positives for any given protein family in which only a subset of its members are involved in immune pathways. For example, sixty-four of the innate immunity genes in the original *Nasonia* genome annotation are not found in our annotation using the IIID; these genes are classified as serine proteases. Several serine proteases play important roles in insect innate immune pathways, specifically the Toll pathway and the prophenoloxidase signaling cascade leading to melanization [17], [27]–[32]. However, the serine protease family is highly diverse, and most of its members function in other aspects of insect physiology [33]–[36]. A HMM that identifies conserved serine protease domains may simply find any serine protease, regardless of its biological function or relevance to insect immunity. Using the IIID for sequence similarity searches partially avoids this source of error because the search is performed using an entire gene, not just a protein domain, which has been identified as part of the innate immune system in another insect species. For example, the IIID predictions identified only 38 serine proteases while the HMMs found 97 serine proteases. Nevertheless, further experimental approaches are needed to determine whether the genes that we have identified actually function in the *Nasonia* immune system.

The other obvious limitation of using a sequence similarity based approach to find immune genes in a specific gene set is that the analysis misses any species-specific genes. For example, thirty-nine genes from our manual search of the literature (that were not detected by the BLASTx analysis) are antimicrobial peptides (AMPs) unique to the *Nasonia* genus, which were predicted computationally based on structural properties common to AMPs [24], [25]. Sequence similarity searches are also constrained by the reference species used to generate the database. Genes in the *Nasonia* immune repertoire present in an insect species not in the IIID would also be missed, although they are not unique to *Nasonia.*

In total, 489 unique genes have been described as potential immune genes in *N. vitripennis* (Table S2) when all previously published studies [16], [24]–[26], manual annotations, and sequence similarity searches using the IIID are combined. To our knowledge, this list is the most complete set of insect immunity genes currently available and the first to include those from *N. giraulti* and *N. longicornis*. While future studies are needed to confirm the functionality of these genes in the *Nasonia* immune response, the list will provide a stepping-stone for comparative analyses within the *Nasonia* genus and between *Nasonia* and other insect species. More importantly, the IIID will provide one more tool in the efforts to annotate complete immune gene repertoires in other insect genomes. Based on our investigation, we recommend the use of multiple annotation tools that will provide the most comprehensive set of predictions *in silico*, which can then be analyzed for their biological role *in vivo*.

## Supporting Information

Table S1 **Top BLASTx results for** ***Nasonia vitripennis*** **against the four genomes in the IIID.**
(XLSX)

Table S2 **A complete list of** ***Nasonia vitripennis*** **putative immunity genes reported in the IIID.**
(XLSX)

Table S3 **A complete list of** ***Nasonia giraulti*** **putative immunity genes reported in the IIID.**
(XLSX)

Table S4 **A complete list of** ***Nasonia longicornis*** **putative immunity genes reported in the IIID.**
(XLSX)

## References

[1] Cooper MD, Alder MN (2006) The evolution of adaptive immune systems. Cell 124: 815–822. 16497590 10.1016/j.cell.2006.02.001

[2] Beckage NE (2008) Insect Immunoloty. Oxford: Academic Press.

[3] Loker ES, Adema CM, Zhang SM, Kepler TB (2004) Invertebrate immune systems–not homogeneous, not simple, not well understood. Immunol Rev 198: 10–24. 15199951 10.1111/j.0105-2896.2004.0117.x PMC5426807

[4] Lazzaro BP, Little TJ (2009) Immunity in a variable world. Philosophical Transactions of the Royal Society B-Biological Sciences 364: 15–26. 10.1098/rstb.2008.0141 PMC2666692 18926975

[5] Robinson GE, Hackett KJ, Purcell-Miramontes M, Brown SJ, Evans JD, et al (2011) Creating a buzz about insect genomes. Science 331: 1386. 21415334 10.1126/science.331.6023.1386

[6] Bordenstein SR, Werren JH (2007) Bidirectional incompatibility among divergent *Wolbachia* and incompatibility level differences among closely related Wolbachia in *Nasonia* . Heredity 99: 278–287. 17519968 10.1038/sj.hdy.6800994

[7] Chafee ME, Zecher CN, Gourley ML, Schmidt VT, Chen JH, et al (2011) Decoupling of host-symbiont-phage coadaptations following transfer between insect species. Genetics 187: 203–215. 20944019 10.1534/genetics.110.120675 PMC3018312

[8] Brucker RM, Bordenstein SR (2012) The roles of host evolutionary relationships (genus: *Nasonia*) and development in structuring microbial communities. Evolution 66: 349–362. 22276533 10.1111/j.1558-5646.2011.01454.x

[9] Keller RG, Desplan C, Rosenberg MI (2010) Identification and characterization of *Nasonia* Pax genes. Insect Mol Biol 19 Suppl 1109–120. 20167022 10.1111/j.1365-2583.2009.00921.x PMC2852259

[10] Lynch JA, El-Sherif E, Brown SJ (2012) Comparisons of the embryonic development of *Drosophila*, *Nasonia*, and *Tribolium* . Wiley Interdisciplinary Reviews: Developmental Biology 1: 16–39. 23801665 10.1002/wdev.3 PMC5323069

[11] Loehlin DW, Werren JH (2012) Evolution of shape by multiple regulatory changes to a growth gene. Science 335: 943–947. 22363002 10.1126/science.1215193 PMC3520604

[12] Clark ME, O'Hara FP, Chawla A, Werren JH (2010) Behavioral and spermatogenic hybrid male breakdown in *Nasonia* . Heredity (Edinb) 104: 289–301. 20087395 10.1038/hdy.2009.152 PMC2872237

[13] Desjardins CA, Perfectti F, Bartos JD, Enders LS, Werren JH (2010) The genetic basis of interspecies host preference differences in the model parasitoid *Nasonia* . Heredity (Edinb) 104: 270–277. 20087393 10.1038/hdy.2009.145 PMC2823958

[14] Niehuis O, Bullesbach J, Judson AK, Schmitt T, Gadau J (2011) Genetics of cuticular hydrocarbon differences between males of the parasitoid wasps *Nasonia giraulti* and *Nasonia vitripennis* . Heredity (Edinb) 107: 61–70. 21179062 10.1038/hdy.2010.157 PMC3186122

[15] Blaul B, Ruther J (2011) How parasitoid females produce sexy sons: a causal link between oviposition preference, dietary lipids and mate choice in *Nasonia* . Proceedings of the Royal Society B: Biological Sciences 278: 3286–3293. 21429922 10.1098/rspb.2011.0001 PMC3169019

[16] Werren JH, Richards S, Desjardins CA, Niehuis O, Gadau J, et al (2010) Functional and evolutionary insights from the genomes of three parasitoid *Nasonia* species. Science 327: 343–348. 20075255 10.1126/science.1178028 PMC2849982

[17] Waterhouse RM, Kriventseva EV, Meister S, Xi Z, Alvarez KS, et al (2007) Evolutionary dynamics of immune-related genes and pathways in disease-vector mosquitoes. Science 316: 1738–1743. 17588928 10.1126/science.1139862 PMC2042107

[18] De Gregorio E, Spellman PT, Rubin GM, Lemaitre B (2001) Genome-wide analysis of the *Drosophila* immune response by using oligonucleotide microarrays. Proc Natl Acad Sci U S A 98: 12590–12595. 11606746 10.1073/pnas.221458698 PMC60098

[19] Obbard DJ, Welch JJ, Kim KW, Jiggins FM (2009) Quantifying adaptive evolution in the *Drosophila* immune system. PLoS Genet 5: e1000698. 19851448 10.1371/journal.pgen.1000698 PMC2759075

[20] Parmakelis A, Moustaka M, Poulakakis N, Louis C, Slotman MA, et al (2010) *Anopheles* immune genes and amino acid sites evolving under the effect of positive selection. PLoS One 5: e8885. 20126662 10.1371/journal.pone.0008885 PMC2811201

[21] Evans JD, Aronstein K, Chen YP, Hetru C, Imler JL, et al (2006) Immune pathways and defence mechanisms in honey bees *Apis mellifera* . Insect Mol Biol 15: 645–656. 17069638 10.1111/j.1365-2583.2006.00682.x PMC1847501

[22] Gerardo NM, Altincicek B, Anselme C, Atamian H, Barribeau SM, et al (2010) Immunity and other defenses in pea aphids, *Acyrthosiphon pisum* . Genome Biol 11: R21. 20178569 10.1186/gb-2010-11-2-r21 PMC2872881

[23] Christophides GK, Zdobnov E, Barillas-Mury C, Birney E, Blandin S, et al (2002) Immunity-related genes and gene families in *Anopheles gambiae* . Science 298: 159–165. 12364793 10.1126/science.1077136

[24] Tian C, Gao B, Fang Q, Ye G, Zhu S (2010) Antimicrobial peptide-like genes in *Nasonia vitripennis*: a genomic perspective. BMC Genomics 11: 187. 20302637 10.1186/1471-2164-11-187 PMC2853521

[25] Tian C, Wang L, Ye G, Zhu S (2010) Inhibition of melanization by a *Nasonia* defensin-like peptide: implications for host immune suppression. J Insect Physiol 56: 1857–1862. 20708012 10.1016/j.jinsphys.2010.08.004

[26] Ye J, Zhao H, Wang H, Bian J, Zheng R (2010) A defensin antimicrobial peptide from the venoms of *Nasonia vitripennis* . Toxicon 56: 101–106. 20362606 10.1016/j.toxicon.2010.03.024

[27] Ligoxygakis P, Pelte N, Hoffmann JA, Reichhart JM (2002) Activation of *Drosophila* Toll during fungal infection by a blood serine protease. Science 297: 114–116. 12098703 10.1126/science.1072391

[28] Jang IH, Chosa N, Kim SH, Nam HJ, Lemaitre B, et al (2006) A Spatzle-processing enzyme required for toll signaling activation in *Drosophila* innate immunity. Developmental cell 10: 45–55. 16399077 10.1016/j.devcel.2005.11.013

[29] Katsumi Y, Kihara H, Ochiai M, Ashida M (1995) A serine protease zymogen in insect plasma. Purification and activation by microbial cell wall components. European journal of biochemistry/FEBS 228: 870–877. 10.1111/j.1432-1033.1995.tb20334.x 7737188

[30] Leclerc V, Pelte N, El Chamy L, Martinelli C, Ligoxygakis P, et al (2006) Prophenoloxidase activation is not required for survival to microbial infections in *Drosophila* . EMBO reports 7: 231–235. 16322759 10.1038/sj.embor.7400592 PMC1369246

[31] Tang H, Kambris Z, Lemaitre B, Hashimoto C (2006) Two proteases defining a melanization cascade in the immune system of *Drosophila* . The Journal of biological chemistry 281: 28097–28104. 16861233 10.1074/jbc.M601642200

[32] Zou Z, Shin SW, Alvarez KS, Kokoza V, Raikhel AS (2010) Distinct melanization pathways in the mosquito *Aedes aegypti* . Immunity 32: 41–53. 20152169 10.1016/j.immuni.2009.11.011

[33] Chasan R, Anderson KV (1989) The role of easter, an apparent serine protease, in organizing the dorsal-ventral pattern of the Drosophila embryo. Cell 56: 391–400. 2492450 10.1016/0092-8674(89)90242-0

[34] Moussian B, Roth S (2005) Dorsoventral axis formation in the *Drosophila* embryo–shaping and transducing a morphogen gradient. Current biology : CB 15: R887–899. 16271864 10.1016/j.cub.2005.10.026

[35] Muhlia-Almazan A, Sanchez-Paz A, Garcia-Carreno FL (2008) Invertebrate trypsins: a review. Journal of comparative physiology B, Biochemical, systemic, and environmental physiology 178: 655–672. 10.1007/s00360-008-0263-y 18404270

[36] Schneider DS, Jin Y, Morisato D, Anderson KV (1994) A processed form of the Spatzle protein defines dorsal-ventral polarity in the *Drosophila* embryo. Development 120: 1243–1250. 8026333 10.1242/dev.120.5.1243

